# 66919 Third trimester electronic cigarette use and the risk of preterm birth, low birthweight, and small-for-gestational age

**DOI:** 10.1017/cts.2021.714

**Published:** 2021-03-30

**Authors:** Lin Ammar, Pingsheng Wu

**Affiliations:** 1 Vanderbilt University Masters of Public Health Program; 2 Vanderbilt University Medical Center

## Abstract

ABSTRACT IMPACT: Our study suggests that maternal e-cigarette use may reduce fetal growth and pose harm to newborns. OBJECTIVES/GOALS: Women are motivated to quit smoking during pregnancy. Many view electronic cigarettes (e-cigarettes) as a safer and healthier alternative to traditional tobacco smoke. We aim to determine the effect of third-trimester e-cigarette use on the risk of infant related outcomes. METHODS/STUDY POPULATION: We conducted a cross-sectional survey study using Pregnancy Risk Assessment Monitoring System (PRAMS). Women who gave live singleton births in 2016-2018 and at states that met response rate threshold criteria were included. Women were classified as never smokers, sole e-cigarette smokers, sole traditional cigarette smokers, and dual-users. Logistic regression was conducted to determine the association between third-trimester cigarette use and preterm birth (<37 weeks), low birth weight (<2,500 grams), and small for gestational age births (SGA, weight lower than the tenth percentile of the population). Analyses were weighted to account for the survey design and non-response. RESULTS/ANTICIPATED RESULTS: 94,539 women (weighted population of 4,765,290) were included. Compared with never smokers, third-trimester sole e-cigarette use increased the odds of preterm birth (Adjusted odds ratio [AOR]: 1.61, 95% confidence interval [CI]: 1.05, 2.48), low birthweight (AOR: 1.49, 95%CI: 1.06, 2.09), and SGA (AOR: 1.19, 95%CI: 0.71, 2.00), sole traditional cigarette use increased the odds of preterm birth (AOR: 1.36, 95%CI: 1.21, 1.52), low birthweight (AOR: 1.90, 95%CI: 1.72, 2.10), and SGA (AOR: 2.28, 95%CI: 2.05, 2.53); and dual use increased the odds of preterm birth (AOR: 1.17, 95%CI: 0.82, 1.67), low birthweight (AOR: 2.16, 95%CI: 1.58, 2.96), and SGA (AOR: 2.67, 95%CI: 1.97, 3.64). DISCUSSION/SIGNIFICANCE OF FINDINGS: E-cigarette use, by itself or in combination with traditional cigarettes, increases the risk of preterm birth, low birthweight, and SGA. Our study suggests that maternal e-cigarette use may reduce fetal growth and pose harm to newborns.

